# The impact of post-stroke fatigue on inpatient rehabilitation outcomes: An observational study

**DOI:** 10.1371/journal.pone.0302574

**Published:** 2024-05-31

**Authors:** Hongji Zeng, Jiaying Yang, Junfa Wu, Yu Ding, Shuya Yuan, Rui Wang, Weijia Zhao, Xi Zeng

**Affiliations:** 1 School of Public Health, Zhengzhou University, Zhengzhou, China; 2 Department of Rehabilitation Medicine, The First Affiliated Hospital of Zhengzhou University, Zhengzhou, China; 3 Department of Rehabilitation Medicine, Huashan Hospital, Fudan University, Shanghai, China; 4 National Center for Neurological Disorders, Shanghai, China; 5 Department of Neurology, The Second Medical Center, PLA General Hospital, Beijing, China; 6 NHC Key Laboratory of Prevention and Treatment of Cerebrovascular Diseases, Zhengzhou, China; Transilvania University of Brasov: Universitatea Transilvania din Brasov, ROMANIA

## Abstract

**Background:**

Post-stroke fatigue is a typical complication following stroke. However, existing research primarily focused on its underlying mechanisms, and its impact on rehabilitation outcomes has yet to be uncovered.

**Objective:**

This study aims to explore the impact of post-stroke fatigue on rehabilitation outcomes during hospitalization.

**Method:**

This was a prospective multicenter observational study including 46 stroke patients receiving comprehensive rehabilitation treatment. Patients’ basic information was recorded upon admission and patients’ functional independence was assessed with Functional Independence Measure (FIM) both upon admission and discharge. One week after rehabilitation treatment, fatigue, positivity in daily activity, attention, and memory were assessed. Serum biochemical indicators and levels of C-reactive protein (CRP) were assessed weekly following admission. The pain scores were assessed during the first week of hospitalization to calculate the average. Correlation analysis, linear regression and propensity score matching (PSM) were used to analyze the impact of fatigue on FIM scores at discharge and length of hospital stay.

**Result:**

The proportion of patients with low fatigue was 39.13% and significant improvement was revealed in FIM scores upon admissions and discharge [(50.67±18.61) vs. (75.13±21.04), P<0.05]. Positivity in daily activity, attention, and age are factors that influence post-stroke fatigue. After PSM, low-fatigue group (Fatigue score< 3) showed significant higher motor function independence at discharge [(54.39 ± 15.42) vs. (41.89 ± 14.90), P<0.05] and shorter hospital stay [(28.54±9.13)d vs. (37.32 ± 9.81)d, P<0.05] than high-fatigue group. There was a significant difference (P<0.05) in level of CRP between the first inpatient week and the third week, with declining trend.

**Conclusion:**

Post-stroke fatigue can affect the rehabilitation outcomes regarding motor function independence and length of hospital stay.

## 1. Introduction

Stroke is a prevalent disease that threatens the health and quality of life of middle-aged and elderly individuals [1]. The high prevalence, disability, and mortality associated with stroke impose a substantial burden on both families and society [2]. In China, there are 1.5–2 million new stroke cases reported annually, with over 7 million survivors, 70% of whom experience varying degrees of disability [3]. Furthermore, stroke frequently presents with various complications such as post-stroke depression, vascular cognitive impairment, and post-stroke fatigue [4]. Among them, post-stroke fatigue is characterized by persistent feelings of exhaustion and weakness following mental or physical activities, which cannot be alleviated by rest [5]. Studies have reported that the prevalence of post-stroke fatigue during the recovery period ranges from 29% to 68% [6]. Post-stroke fatigue not only hampers patients’ daily activities but also serves as an independent risk factor affecting their quality of life and social participation [7].

Rehabilitation treatment is extremely important for the prognosis of stroke patients [8]. Comprehensive and standardized rehabilitation therapy can not only alleviate the disability burden, improve physical function, but also reduce the incidence of complications and risk of recurrent stroke [8]. Therefore, stroke survivors are generally transferred to the Department of Rehabilitation Medicine for further treatment once their condition stabilizes [9]. However, a decline in physical function and changes in psychological factors can often lead to post-stroke fatigue [7]. Fatigue can adversely affect rehabilitation compliance, undermine confidence in the rehabilitation process, and even exacerbate negative emotions, thereby prolonging the overall recovery journey [10].

Based on this, we speculated that post-stroke fatigue may lead to poor rehabilitation outcomes. Although there is extensive research on the factors influencing post-stroke fatigue, there remains a lack of specific studies examining its precise influence on rehabilitation outcomes during hospitalization. Clinical practice should be formulated based on an accurate study of the association between post-stroke fatigue and rehabilitation effect [11]. It is evident that insufficient specific research on post-stroke fatigue may lead to incomplete understanding, resulting in a failure to improve patients’ conditions in a targeted manner. Therefore, in this study, we collected data on functional assessment and relevant biochemical indicators of stroke patients during their rehabilitation hospitalization, aiming to explore whether post-stroke fatigue affects the effectiveness of rehabilitation treatment during hospitalization, in order to provide references for the clinical rehabilitation management of patients with post-stroke fatigue during hospitalization.

## 2. Method

### 2.1 Study participants

This was a prospective multicenter observational study. Before the study began, we conducted the sample size estimation through a literature review [12]. We assumed a type I error probability of α = 0.05, P = 58.90%, and margin of error of 0.2. Based on the reported prevalence of post-stroke fatigue during hospitalization (58.9%), the specific formula was as follows:

$$n={\left(Z_{\alpha /2}\right)}^{2}*[p(1‐p)]/(d^{2})\approx 24$$

Based on an estimated 20% dropout rate, we estimated that at least 29 patients would be required. Next, we began the study in 2022. When we achieved the minimum sample size, we did not stop recruiting until September of the same year.

Totally, 46 patients with stroke from Jan 2022 to December 2022 admitted to the Department of Rehabilitation Medicine of 3 hospitals in China were enrolled. The inclusion criteria were: 1) Age ≥ 18 years; 2) Stable vital signs; 3) Meeting the diagnosis of stroke [13]; 4) Actively participating in rehabilitation training; 5) Transferred or admitted to the Department of Rehabilitation Medicine within two weeks of onset; 6) First-time stroke. The exclusion criteria were: 1) Combined with other neurological disorders or having a history of other neurological disorders; 2) Combined with severe cognitive or communication impairments, unable to complete the questionnaire; 3) Presence of severe psychological disorders; 4) Combined with severe liver, kidney, or other systemic diseases, or diagnosed with tumors. 5) Pregnant or nursing females; 6) Hospitalization in the Department of Rehabilitation Medicine<1 week. The dropout criteria were: 1) a patient or family members requested to withdraw voluntarily; 2) The patient’s condition deteriorated severely.

The study was conducted according to the Declaration of Helsinki and was approved by the Ethics Committee of the First Affiliated Hospital of Zhengzhou University (Ethic number: 2021-ky-1333). Before enrolling in the study, we obtained written informed consent from each patient. The consent included their agreement to use data from their medical records for research purposes. All data were fully anonymized before statistical analysis.

All patients received comprehensive rehabilitation treatment, including risk factor intervention (such as hypertension control, diabetes management, hyperlipidemia control), lifestyle modification (such as balanced diet, exercise, and smoking cessation), medication therapy (such as antiplatelet agents, anticoagulant medications, thrombolytic drugs, and neurorecovery drugs), rehabilitation training (e.g., motor function training, activities of daily living training, sensory function training, language and cognitive function training, and balance and coordination function training), and psychological support. Specific content and frequency were determined according to the patient’s condition [9].

After enrollment, the participants were numbered in the database for privacy. The assessment and data collection were conducted by different staff members who maintained an isolated status from the patients enrolled beyond the necessary contact, strictly adhering to the principle of not disclosing group information. All medical personnel received detailed training to ensure understanding and consistency of the study design. Additionally, the principal investigator regularly supervised the study.

### 2.2 Assessment

#### 2.2.1 Fatigue and positivity in daily activity

This assessment was performed after one week of rehabilitation treatment (on day 8 of hospitalization in the Department of Rehabilitation Medicine).

Fatigue was assessed using the Visual Analog Fatigue Scale (VAS-F). This scale can help patients accurately express their level of fatigue through pictures, with reliable reliability and validity, especially regarding the impact of fatigue on daily life for stroke patients (S1 Appendix). The scale included two subscales: degree of fatigue and impact on positivity in daily activities. The first subscale was set to assess the patient’s degree of fatigue with the question, ‘How tired have you felt over the last week?’ Then, based on the patient’s choice, the score was given ranging from 0 (no tired at all) to 4 (extremely tired), with a total of five levels. With the question "How much does feeling tired prevent you from doing what you want to do?" A score ranging from 0 (no impact at all) to 4 (I can do very little) was given to the second question, with a total of five levels. "As the level rises, Fatigue and its impact on positivity in daily activity also increase." This scale had good retest reliability (Weighted Kappa 0.71 for Question 1 and 0.72 for Question 2) and good equivalence of method (Weighted Kappa 0.64 for Question 1 and 0.65 for Question 2) [14].

Regarding the first question, a fatigue score <3 indicated low fatigue, while a score ≥3 indicated high fatigue.

#### 2.2.2 Basic information

Basic patient information was extracted from the corresponding hospital’s medical record system to ensure completeness and accuracy. These included: 1) Body Mass Index (BMI, to evaluate obesity status); 2) comorbidities (including hypertension, atrial fibrillation, coronary artery disease, and diabetes, all of which were risk factors for stroke), 3) stroke type (ischemic or hemorrhagic), 4) lesion location (left, right, or bilateral), and 5) length of hospital stay (recorded after the patient’s discharge).

#### 2.2.3 Functional independence

This assessment was conducted for each patient upon admission (to the Department of Rehabilitation Medicine) and at discharge. The Functional Independence Measure (FIM), an internationally recognized and important component of the American Rehabilitation Data System, was used to assess functional independence in this study [15]. The FIM comprised a total of 18 items, including 13 motor function assessments (Eating, Grooming, Bathing, Dressing upper body, Dressing lower body, Toileting, Bowel control, Bladder control, Transfer between bed and chair or wheelchair, Transfer in toilet, Transfer in bathroom, Walking or wheelchair moving, Stair activity) and 5 cognitive function assessments (Comprehension—visual, auditory or both, Expression—visual, auditory or both, Social interaction, Problem solving, Memory). Each item was scored from 1 (total dependence) to 7 (complete independence), with a total score ranging from 18 to 126. A higher score indicates a lower level of functional independence.

#### 2.2.4 Memory and attention

This assessment was performed after one week of rehabilitation treatment (on day 8 of hospitalization in the Department of Rehabilitation Medicine).

The assessment of attention included figure, story recall, word learning, and daily life tests. Specifically, in the figure tests, patients were shown a polygon with six sides and were then required to draw it independently. They received a score of 0 if they succeeded, and a score of 1 otherwise. In the story recall tests, an independent staff member told a story of no more than 150 Chinese characters and asked the patient to retell it. If correct, they received 0 points; otherwise, they received 1 point. In the word learning tests, patients were required to memorize the three ordered common item names. They received a score of 0 if they succeeded, and a score of 1 otherwise. In daily life tests, patients were required to indicate the province where they were located, the date of the day, and yesterday’s dinner. If they answered correctly, they received 0 points; otherwise, they received 1 point. The scores from these four components were summed up to determine the final memory assessment score of the patient.

Attention assessment included cancellation tests, auditory concentration tests, continuous performance tests, and subjective ratings. Specifically, the material for the cancellation test was designed to be completed within 60 seconds by a normal individual aged 30 years with an accuracy rate of over 95%. Patients were given 90 seconds to complete the test, and if their accuracy rate was above 60%, they received a score of 0; otherwise, they received a score of 1. In the auditory concentration test, patients were presented with a series of sounds or words and instructed to pay attention to specific sounds. In the continuous performance test, patients faced a computer screen where a continuous series of stimuli (such as shapes, colors, or numbers) were presented, and were required to press the key when specific stimuli appeared. Both the tests were conducted five times. If the patients succeeded three times, they received a score of 0; otherwise, they received a score of 1. The subjective ratings were provided by experienced speech therapists who were independent of the study and the patient’s treatment process. They observed the patients’ performance during all tests and assigned a score. A score of 0 indicated normal attention, whereas a score of 1 indicated impaired attention. The scores from these four components were summed up to determine the patient’s final attention assessment score.

Each part of the assessment of memory and attention was commonly used in clinical practice, with good reliability and validity [16]. However, due to limitations inherent in each part, they were combined for use in this study.

#### 2.2.5 Serum biochemical data

Serum biochemistry data, including blood urea nitrogen (BUN, mg/dL), aspartate aminotransferase (AST, units/L), alanine aminotransferase (ALT, units/L), white blood cells (WBC, K/mm3), red blood cells (RBC, M/uL), and hematocrit (HCT, %), were collected weekly in the corresponding hospital’s medical record system after the patient’s admission. The mean values during hospitalization were calculated based on weekly data.

#### 2.2.6 C-reactive protein

The patient’s serum was collected weekly, and the C-reactive protein (CRP) level was determined using a human CRP immunoassay Enzyme-Linked Immunosorbent Assay (ELISA) kit. The procedure was shown as follows.

1. Add 100μL of Detection Diluent RD1F to each well of the microplate and shake gently.
2. Add 50μL of the Standard Solution, Diluted Standard Solution, or sample serum to each well in sequence. The plate was then covered with a film and incubated at room temperature for 2 hours.
3. Discard the liquid from the microplate and wash each well three times with 400μL of Wash Buffer.
4. Add 200μL of CRP Conjugate to each well. Cover with a new film and incubate at room temperature for 2 hours.
5. Discard the liquid from the microplate and wash each well three times with 400μL of Wash Buffer.
6. Add 200μL of Substrate Solution to each well and incubate at room temperature, protected from light, for 30 minutes.
7. Add 50μL of Stop Solution to each well. The blue liquid in the microplate will turn yellow.
8. Measure the optical density at 450nm using an ELISA reader within 30 minutes.
9. Calculate the standard curve and the CRP levels (ng/mL) in the sample serum.

#### 2.2.7 Pain scores

Pain scores were assessed using the visual analog scale (S2 Appendix), in which patients could select corresponding words and images to indicate their pain levels. Pain scores were recorded daily during the first week of hospitalization, and the average score for the 7-day period was calculated.

### 2.3 Statistical analysis

Continuous variables were expressed as mean ± standard deviation, while categorical variables were presented as counts and percentages. The association between fatigue and various variables was analyzed using the Spearman correlation analysis. Paired t-tests were used to analyze the differences in FIM scores upon admission and at dis charge in all samples. Independent sample t-tests were used to analyze the differences in FIM scores between patients with low fatigue (Fatigue score <3) and those with high fatigue (Fatigue score ≥3). Statistically significant variables identified in the univariate analysis were included in linear regression analysis. Propensity score matching (PSM) was used to minimize the potential bias caused by confounding variables. It was used to analyze the differences in rehabilitation outcomes between the two groups [17]. The basic information and variables that exhibited significant correlations with fatigue were selected as the set of covariates. The length of hospital stay was not included in the set of covariates because it was considered indicative of rehabilitation outcomes. Patient fatigue grouping was used as the independent variable, whereas variables regarding rehabilitation outcomes (length of hospital stay, FIM upon admission, and at discharge) were used as dependent variables for matching. A probit regression model was used to estimate the propensity scores for the low- and high-fatigue groups. Paired sample t-tests were used to analyze the trend of CRP changes in the patients. A P-value<0.05 indicated statistical significance, and all data were analyzed using SPSS 21.0.

## 3. Result

### 3.1 Basic information of the patients

A total of 46 stroke patients participated in this study, including 25 males and 21 females with no dropouts. The average age was (69.70±10.27) years. The majority of the participants (82.6%) were diagnosed with ischemic stroke (Table 1).

**Table 1:** Basic information.

Variables	All sample (n = 46)	Hospital A (n = 22)	Hospital B (n = 16)	Hospital C (n = 8)
Age (years, x±s, span)	69.70±10.27, 47–85	68.29±9.94, 47–75	71.10±10.32, 51–69	70.78±10.39. 56–85
Gender (n. %)				
Male	25 (54.35)	14 (63.64)	6 (37.50)	5 (62.50)
Female	21 (45.65)	8 (36.36)	10 (62.50)	3 (37.50)
Length of hospital stay (days, x±s, span)	32.28±12.45, 20–75	26.39±12.29, 20–61	31.93±13.47, 26–58	49.21±7.21, 43–75
BMI (kg/m^2^, x±s, span)	27.75±5.45, 18.41–46.98	27.68±5.86, 19.97–46.98	27.73±5.01, 22.35–32.93	28.01±4.97, 18.41–35.34
Risk factors (n. %)				
Hypertension	39 (84.78)	18 (81.82)	15 (87.50)	6 (75.00)
Atrial fibrillation	11 (23.91)	7 (31.82)	4 (36.36)	-
Coronary artery disease	12 (26.09)	5 (22.73)	5 (31.25)	2 (25.00)
Diabetes	17 (39.96)	10 (45.45)	4 (25.00)	2 (25.00)
Stroke type (n. %)				
Ischemic	38 (82.61)	17 (77.27)	15 (93.75)	6 (75.00)
Hemorrhagic	8 (17.39)	5 (22.73)	1 (6.25)	2 (25.00)
Lesion location (n. %)				
Left	22 (47.82)	9 (40.91)	8 (50.00)	5 (62.50)
Right	24 (52.17)	13 (59.09)	8 (50.00)	3 (37.50)
Bilateral	-	-	-	-

### 3.2 Fatigue and positivity in daily activity

**Fig 1:**
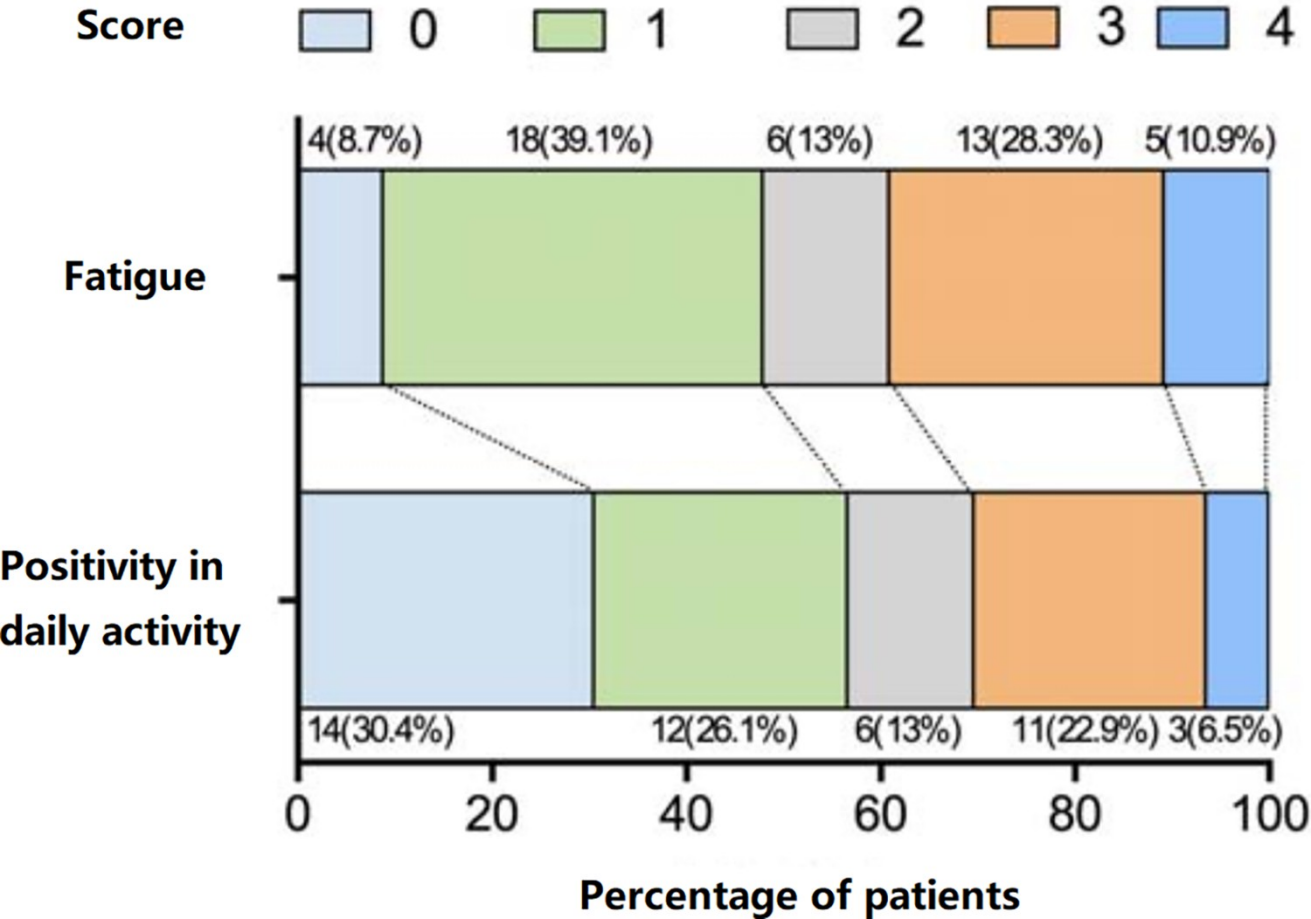
Distribution of fatigue and positivity in daily activity.

The distribution of fatigue and positivity in daily activities were shown in Fig 1. Among the participants, 18 (39.13%) were in the high-fatigue group (fatigue score ≥3) and 28 (61.87%) were in the low-fatigue group (fatigue score <3).

**Fig 2:**
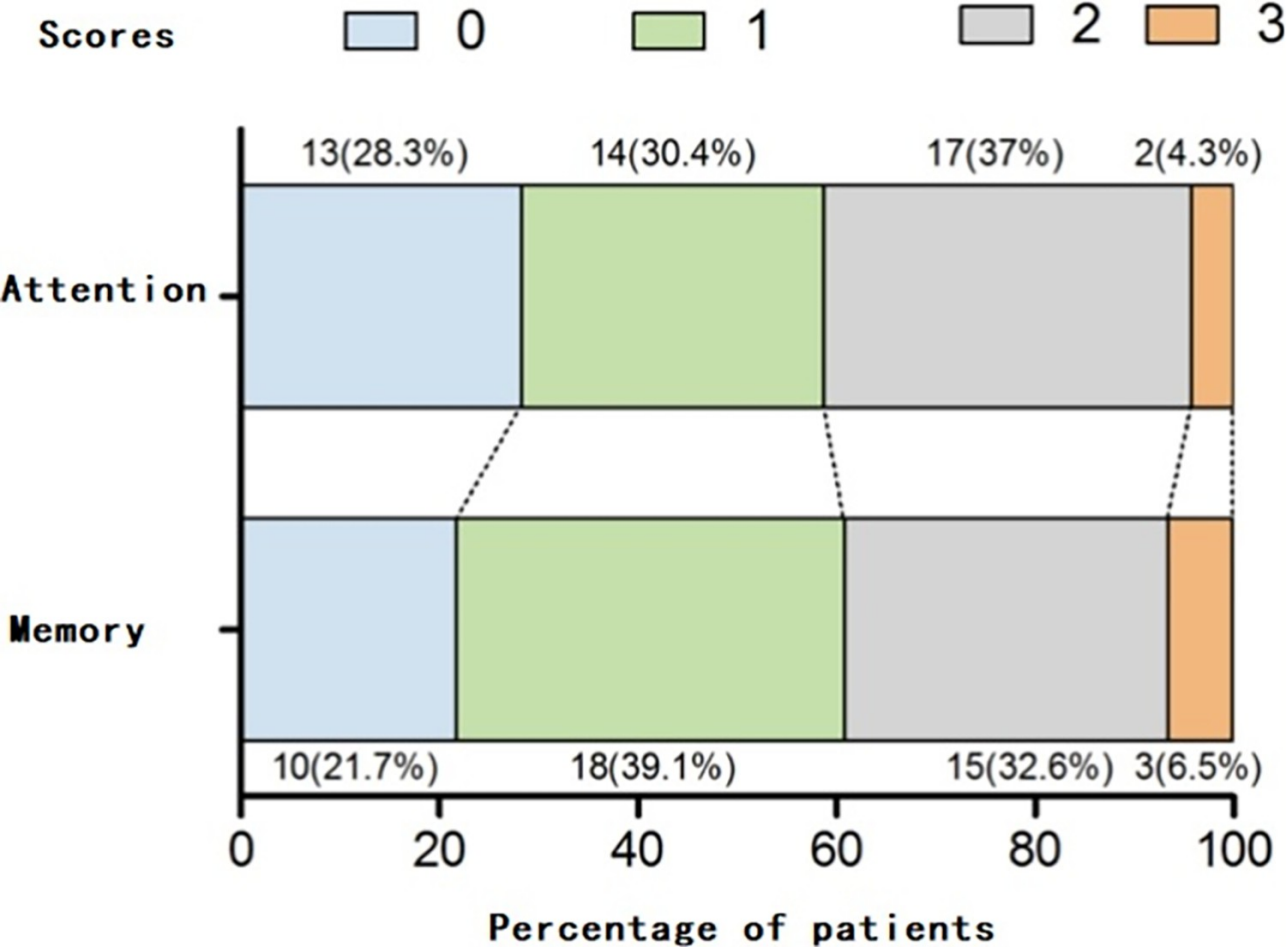
Distribution of memory and attention scores.

The distributions of the memory and attention scores were shown in Fig 2. The details of the patients’ FIM scores, hematological test results, and pain scores were shown in Table 2. The total FIM score upon admission was (50.67±18.61), which increased to (75.13±21.04) upon discharge, with a significant difference between the two assessments(P<0.001).

**Table 2:** Clinical data of the patients upon admission (x ± s).

Variables	
FIM upon admission	
Motor function	30.52±13.19
Cognitive function	18.09±6.56
Total	50.67±18.61
FIM at discharge	
Motor function	47.85±16.50
Cognitive function	24.00±5.39
^a^ Total	75.13±21.04
BUN (mg/dL)	21.60±9.73
AST (units/L)	24.67±8.21
ALT (units/L)	30.92±7.47
WBC (K/mm3)	8.07±2.58
RBC (M/uL)	4.28±0.58
HCT (%)	37.73±4.74
Pain scores	2.04±0.78

^a^ Difference in FIM upon admission and discharge: t = -5.906, p<0.001

### 3.3 Correlation analysis

Spearman correlation analysis results indicated that fatigue was significantly correlated (P<0.05) with positivity in daily activity, attention, age, length of hospital stay, and functional independence. However, there was no significant correlation (P>0.05) between fatigue and memory or cognitive function (Table 3).

**Table 3:** Correlation of fatigue with clinical data.

Variables	r	P
Positivity in daily activity	0.671	<0.001***
FIM upon admission		
Motor function	-0.283	0.057
Cognitive function	-0.123	0.416
Total	-0.253	0.089
FIM at discharge		
Motor function	-0.306	0.039*
Cognitive function	-0.100	0.510
Total	-0.292	0.045*
Attention	0.428	0.003**
Memory	0.179	0.235
Age	0.338	0.022*
Length of hospital stay	0.304	0.040*
BMI	-0.017	0.913
BUN	0.343	0.020*
HCT	-0.334	0.023*
CRP (The first week)	-0.048	0.765
Pain scores	-0.036	0.810

*P<0.05

**P<0.01

***P<0.001

Based on the correlation analysis, variables with statistical significance were selected as independent variables, and fatigue was chosen as the dependent variable. Linear regression analysis was conducted, as shown in Table 4. Since FIM and length of hospital stay were used as dependent variables in the PSM analysis, they were not included in the regression analysis. The linear analysis results indicated that positivity in daily activity, attention, and age were influencing factors of post-stroke fatigue (P < 0.05).

**Table 4:** Regression analysis.

Variables	B	Beta	P	VIF
Positivity in daily activity	0.837	0.673	<0.001***	1.148
Attention	0.549	0.395	0.006**	1.674
Age	0.487	0.301	0.018*	1.517
BUN	0.392	0.106	0.096	1.032
HCT	-0.403	-0.124	0.085	1.776

B: Non standardized coefficient; Beta: Standardized coefficient; VIF: Variance Inflation Factor

*P<0.05

**P<0.01

***P<0.001

### 3.4 PSM analysis on the impact of fatigue on rehabilitation outcomes

Of the 46 samples, 36 were matched using PSM. After matching, there were no statistically significant differences (p>0.05) in basic information, positivity in daily activity, attention, or age among the participants. The kernel density plot and comparison of the standardized bias before and after PSM were shown in the S3 Appendix.

After PSM, there was no significant difference (P > 0.05) in the functional independence scores between the high-fatigue group (fatigue score ≥ 3) and low-fatigue group (fatigue score < 3) upon admission. Parentheses were added after the data of each sub-variable under FIM at discharge to indicate the p-values of group differences compared with before treatment. After rehabilitation treatment, both groups showed significant improvements in all the sub-variables under the FIM. However, the low-fatigue group demonstrated significantly better motor functional independence than the high-fatigue group (P < 0.05). There was no significant difference in cognitive functional independence and total FIM scores between the two groups before and after rehabilitation treatment (P>0.05). The low-fatigue group had a significantly shorter length of hospital stay than the high-fatigue group (P < 0.05). All details were shown were shown in Table 5.

**Table 5:** Comparison of FIM between low-fatigue group and high-fatigue group (x±s).

Variables	low-fatigue group (n = 18)	high-fatigue group (n = 18)	P
FIM upon admission			
Motor function	29.71 ± 12.02	26.78 ± 13.11	0.489
Cognitive function	18.12 ± 6.57	16.89 ± 6.31	0.571
Total	47.83 ± 16.98	45.44 ± 19.30	0.696
FIM at discharge			
Motor function	54.39 ± 15.42 (P<0.001***)	41.89 ± 14.90 (P = 0.003**)	0.018*
Cognitive function	24.68 ± 5.41 (P = 0.002**)	22.89 ± 6.44 (P = 0.008**)	0.373
Total	79.07 ± 17.32 (P<0.001***)	67.78 ± 21.10 (P = 0.002**)	0.094
Length of hospital stay	28.54±9.13	37.32 ± 9.81	0.009**

*P<0.05

**P<0.01

***P<0.001

### 3.5 Levels of CRP

**Fig 3:**
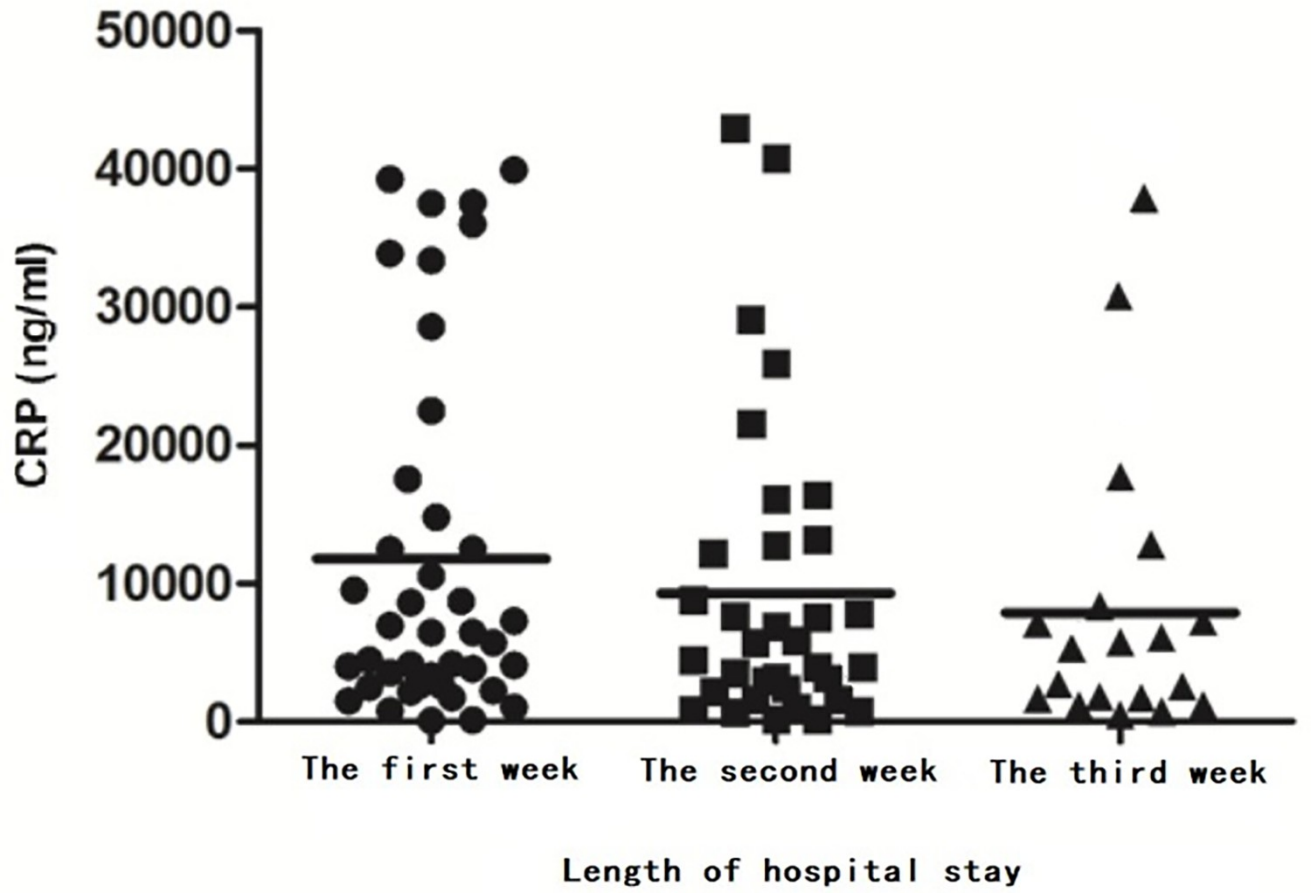
Trend in changes of levels of CRP.

Before PSM, Spearman’s correlation analysis showed no significant association between CRP levels in the first week and fatigue. After PSM, there was no significant association between average CRP levels during the first three weeks of hospitalization and fatigue (P > 0.05). The paired t-test analysis of levels CRP during the first three weeks of hospitalization showed a decreasing trend in CRP levels during hospitalization (Fig 3), with a significant decrease in CRP levels in the first week compared to the third week (P = 0.007).

## 4. Discussion

Post-stroke fatigue is a frequent and potential complication during the recovery period in patients. A study conducted in China revealed a prevalence of approximately 60% [18], which is consistent with the findings of the current study. However, an Australian meta-analysis revealed a prevalence of approximately 50% [19], slightly lower than the 61.87% reported in this study. This difference may be attributed to variations in the regions, criteria, and timing of assessments across studies. Rehabilitation therapy during the post-stroke hospitalization period is crucial for improving patient prognosis. However, physical decline and changes in mental state can easily contribute to the development of post-stroke fatigue [7]. Therefore, investigating the impact of post-stroke fatigue on rehabilitation outcomes holds practical significance.

This study revealed a significant correlation between fatigue and motor function independence, consistent with a previous study in the United States [20]. Based on this, the study included the variable of positivity in daily activities. This can help to elucidate the potential psychological mediating factors between fatigue and motor function independence. Notably, the low-fatigue group exhibited greater improvement in motor FIM scores at discharge compared to the high-fatigue group. Post-stroke fatigue may lead to a decrease in patients’ limb motor activity, thus reducing their independence in motor functions. Similarly, it may result in a decrease in positivity in daily activities, characterized by feelings of fatigue and reduced initiative in engaging in activities [21]. This can diminish patients’ willingness and capacity to participate in rehabilitation activities, resulting in reduced engagement [22]. Research by Lenze et al. indicated that low participation in rehabilitation training among patients with post-stroke fatigue delayed limb function recovery and prolonged hospital rehabilitation duration [23], which partially supports the current study. In addition, although stroke patients often experience cognitive decline due to impaired neurological function, which may exacerbate their sense of fatigue [24], this study found no significant correlation between fatigue and cognitive FIM scores. Similarly, Halvor et al. conducted cognitive assessments on stroke patients using the Mini-Mental State Examination and obtained comparable results [25]. A review suggested that fatigue can reduce the duration of a patient’s ability to concentrate, it may not directly impact cognitive function, offering a partial explanation for these results [26].

To better comprehend the specific mechanisms of post-stroke fatigue, subdividing cognitive functions can be insightful. Wu et al. reviewed 10 studies investigating the relationship between cognition and post-stroke fatigue and found that while cognition was associated with post-stroke fatigue in 8 studies, there was no significant correlation [27]. Building on this research, the current study focused on two variables: attention and memory. The analysis revealed a negative correlation between post-stroke fatigue and attention. In the clinical practice of this study, patient communication highlighted that fatigue hindered their ability to concentrate during rehabilitation exercises, leading to reduced rehabilitation efficiency. Although the exact mechanism underlying the relationship between fatigue and attention remains unclear and larger sample sizes are necessary to confirm the findings’ reliability, the study suggests that intervening in post-stroke fatigue could potentially enhance patients’ attention and improve rehabilitation efficiency.

Most studies on post-stroke fatigue have included age as a variable of interest. However, the relationship between age and post-stroke fatigue remains controversial. This study found a weak association between age and post-stroke fatigue, which is consistent with previous research conducted in New Zealand [28]. On the contrary, Parks et al. found that younger stroke patients were more likely to experience fatigue [29]. Meanwhile, Lerdal et al. discovered that both young patients (age <60 years) and old patients (age ≥75 years) were more prone to post-stroke fatigue compared to those in the middle-aged group (60≤ age <75 years). The study suggested that both young and old patients might suffer more decreased social participation and work ability, increased family life stress, and a rapid decline in physical function due to stroke [30]. Other research has suggested that aging can lead to risk factors for fatigue, such as reduced metabolism and muscle strength [31], loneliness, and depression [32]. These conflicting results may be attributed to differences in social, cultural, and economic conditions across regions. Therefore, the conclusions of this study regarding the relationship between age and post-stroke fatigue should be cautiously generalized to areas outside mainland China. Nonetheless, we recommend paying attention to the age groups more prone to fatigue to reduce the impact on rehabilitation outcomes and improve recovery efficiency.

After controlling for confounding variables, post-stroke fatigue was found to be a significant factor contributing to prolonged hospital stays, which aligns with previous research [33]. Stroke patients typically undergo rehabilitation therapy to regain their functional abilities. However, due to the presence of fatigue, patients may require more time to complete their rehabilitation treatment, consequently leading to an extended duration of hospitalization [22]. Furthermore, post-stroke fatigue can have detrimental effects on psychological well-being, including increased levels of anxiety and depression [34]. These mental health issues can hinder the progress of rehabilitation, further contributing to the prolonged hospital stay.

CRP is a sensitive yet non-specific inflammatory protein that typically remains at low levels in the blood of healthy individuals. However, its concentration rapidly increases during inflammation onset, serving as a marker for systemic inflammation [35]. This study revealed no significant correlation between CRP levels and fatigue. In a long-term study involving 65 patients [36], no correlation was found between CRP levels and post-stroke fatigue at the first and the twelfth month, with only a weak correlation observed at the sixth month (P = 0.04). The factors influencing fatigue may vary across different post-stroke stages, leading to fluctuations in the observed correlations. Qualitative studies have highlighted that during the acute and recovery phases of stroke, lesion location and lifestyle are the primary factors influencing fatigue. As recovery, the direct impact of the stroke on post-stroke fatigue gradually diminishes, and other immune and psychological factors become more prominent [37]. Furthermore, CRP levels in patients significantly decreased during the third week of rehabilitation compared to the first week, and throughout the entire rehabilitation period, a decreasing trend was observed. This suggests that comprehensive rehabilitation training can help reduce inflammatory responses.

There are still controversies in the research on post-stroke fatigue, and this study has potential limitations. First, our sample size was relatively small, and the hospitals enrolled were limited to central China, which may affect the representativeness of the statistical data. In the future, we shall include more patients. Moreover, in this study, fatigue in patients was assessed only once a time, which may have affected the reliability of the data. Additionally, we did not conduct long-term studies to explore the long-term effects of post-stroke fatigue. Finally, although variables such as pain did not show significance in the correlation analysis, their interaction with fatigue may have affected the outcome of rehabilitation. However, this study chose to use PSM to control bias instead of analyzing interactive factors, which remains to be improved in future research.

## 5. Conclusion

Positivity in daily activities, attention, and age are factors that influence post-stroke fatigue. Post-stroke fatigue significantly affects motor function at discharge and length of hospital stay. Therefore, relevant clinical management should be strengthened, and patient fatigue should be regularly monitored for early intervention and treatment.

## Supporting information

S1 Appendix (DOCX)

S2 Appendix (DOCX)

S3 Appendix (DOCX)
