# Correction: The impact of post-stroke fatigue on inpatient rehabilitation outcomes: An observational study

**DOI:** 10.1371/journal.pone.0308750

**Published:** 2024-08-08

**Authors:** The PLOS ONE Staff

The affiliation for the eighth author is incorrect. Xi Zeng not affiliated with #1 but with #2: Department of Rehabilitation Medicine, The First Affiliated Hospital of Zhengzhou University, Zhengzhou, China.

The publisher apologizes for the error.
