# Supplementary figures and images for: The impact of post-stroke fatigue on inpatient rehabilitation outcomes: An observational study
Source: PLoS One. 2024 May 31;19(5):e0302574. doi: 10.1371/journal.pone.0302574 (PMC11142535; doi:10.1371/journal.pone.0302574)

APPENDIX 1


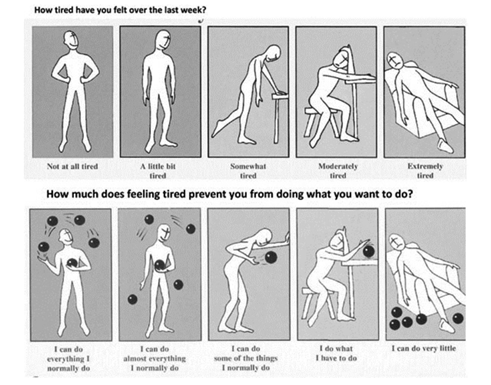

Supplement: S1 Appendix — (DOCX) [file pone.0302574.s001.docx]

APPENDIX 2


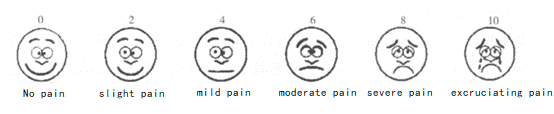

Supplement: S2 Appendix — (DOCX) [file pone.0302574.s002.docx]

APPENDIX 3

Kernel density plot

| Before PSM | After PSM |
| --- | --- |
| 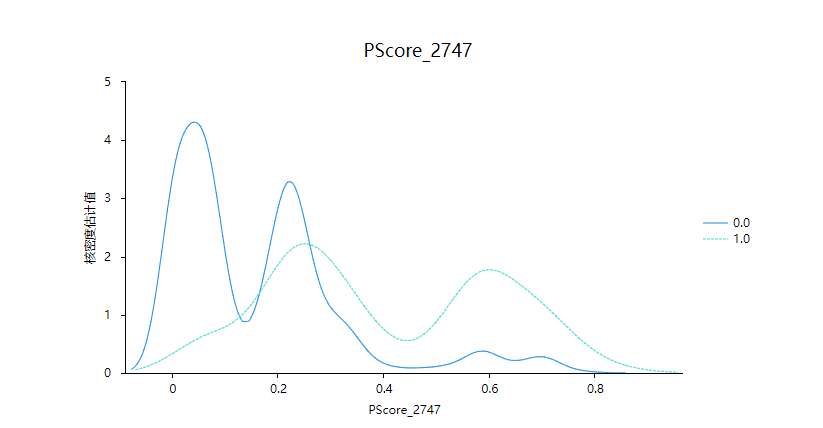 | 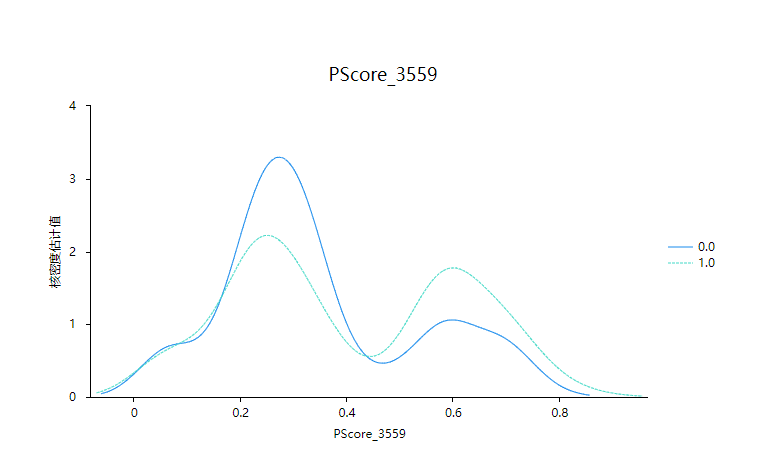 |


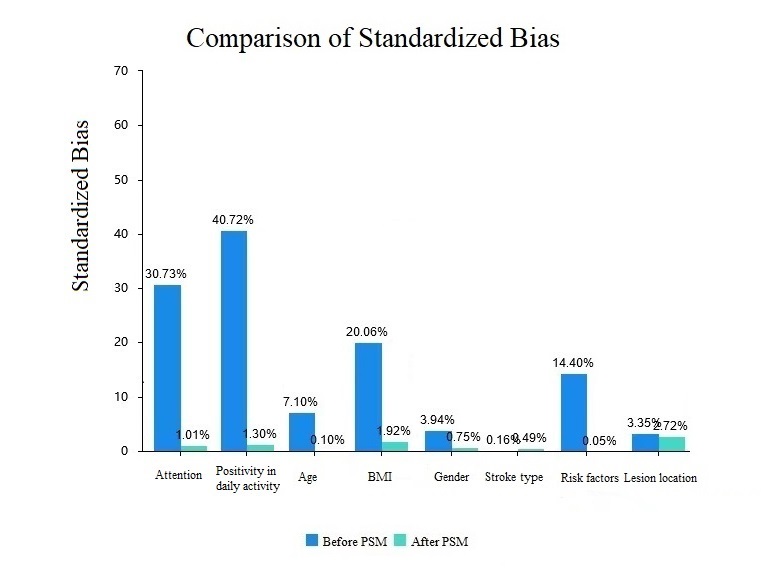

Supplement: S3 Appendix — (DOCX) [file pone.0302574.s003.docx]
